# Argon Ion Implantation as a Method of Modifying the Surface Properties of Wood–Plastic Composites

**DOI:** 10.3390/ma17215347

**Published:** 2024-10-31

**Authors:** Izabela Betlej, Marek Barlak, Karolina Lipska, Piotr Borysiuk, Piotr Boruszewski

**Affiliations:** 1 Department of Wood Science and Wood Protection, Institute of Wood Sciences and Furniture, Warsaw University of Life Sciences—SGGW, 159 Nowoursynowska St., 02-776 Warsaw, Poland; izabela_betlej@sggw.edu.pl; 2 Ion Beam Technology Division, Material Physics Department, National Centre for Nuclear Research Świerk, 7 Sołtana St., 05-400 Otwock, Poland; marek.barlak@ncbj.gov.pl; 3 Department of Technology and Entrepreneurship in Wood Industry, Institute of Wood Sciences and Furniture, Warsaw University of Life Sciences—SGGW, 159 Nowoursynowska St., 02-776 Warsaw, Poland; karolina_lipska@sggw.edu.pl (K.L.); piotr_borysiuk@sggw.edu.pl (P.B.)

**Keywords:** wood–plastic composites, argon ion implantation, polyethylene, lignocellulosic fibres, surface modification, wettability, colour change

## Abstract

Wood–plastic composites (WPCs) combine the properties of plastics and lignocellulosic fillers. A particular limitation in their use is usually a hydrophobic, poorly wettable surface. The surface properties of materials can be modified using ion implantation. The research involved using composites based on polyethylene (PE) filled with sawdust or bark (40%, 50%, and 60%). Their surfaces were modified by argon ion implantation in three fluencies (1 × 10^15^, 1 × 10^16^, and 1 × 10^17^ cm^−2^) at an accelerating voltage of 60 kV. Changes in the wettability, surface energy, and surface colour of the WPCs were analysed. It was shown that argon ion implantation affects the distinct colour change in the WPC surface. The nature of the colour changes depends on the filler used. Implantation also affects the colour balance between the individual variants. Implantation of the WPC surface with argon ions resulted in a decrease in the wetting angle. In most of the variants tested, the most significant effect on the wetting angle changes was the ion fluence of 1 × 10^17^ cm^−2^. Implantation of the WPC surface also increased the surface free energy of the composites. The highest surface free energy values were also recorded for the argon ion fluence of 1 × 10^17^ cm^−2^.

## 1. Introduction

Ion implantation can be a relatively simple and cheap method of the modification of the near-surface regions of different classical materials, like metals and their alloys [1], ceramics [2], semiconductors [3], plastics [4], coatings [5], and more exotic materials, like cellulose, wood, or their composites [6]. This low-temperature method can modify/change the physical and chemical properties of the near-surface regions of solid materials by introducing foreign element ions. The ion beam interacts with the modified material, introduces new atoms, damages its crystal lattice, and creates vacancies and other defects up to total amorphisation. The modified region is not an additional layer. Hence, no adhesion problem occurs (no delamination), and the change in dimensions and the surface finish of the implanted material is negligible [7,8]. The implanted ions form a Gaussian distribution of ions versus depth. The typical value of the range of the implanted ions usually spans from tens to hundreds of nanometers. However, it is sufficient to change the material surface properties, e.g., to increase the tool durability by a factor of 2–3 [9]. The modelling of ion implantation processes allows for the estimation of the ion penetration depth and, thus, the estimation of the thickness of the modified layer.

The ion implantation method is beneficial for modifying the surface properties of materials. Kondyurin et al. [10] showed that Ar ion implantation on the surface of a polystyrene film generates carbonisation, oxidation, and cross-linking effects on the surface layer, which translates into changes in surface roughness and wettability. Sun et al. [11] suggest that these effects are generated by energy transferred through electron excitation or ionisation. The ion implantation method is also helpful for modifying structures occurring in the nanoscale. Narula et al. [12] showed the usefulness of argon ions in modifying the optical and electrical properties of 80 nm diameter CdSe wires. In other studies, Lu et al. [13] proved that silver ion implantation can be successfully used as a method of modifying the surface of polyethylene films to acquire functional properties.

Ion implantation processes can also improve or modify natural materials and their composites. The implantation of nitrogen ions into a composite based on cellulose, silica, and polyvinyl alcohol increased the stiffness of the composite surface and modified its luminescent and thermal properties [14]. In other experiments conducted by Chan et al. [15], it was shown that ion implantation on a cellulose nitrate foil can also be helpful in terms of using such foils in medical diagnostics.

This paper presents the results obtained during the argon ion implantation of two kinds of polyethylene (PE) and a few kinds of wood–plastic composites (WPCs), with a PE matrix and reinforcement in the form of wood (sawdust) or bark. Based on the authors’ previous work [16], we decided to investigate the suitability of argon ion implantation for modifying the WPC surface. WPC composites are a popular material with excellent functional properties and resistance to atmospheric factors. They can be used in various places and under various conditions. 

Due to the content of lignocellulosic particles, the prepared composites may be characterised by higher water absorption, which may translate into a shorter cycle of their use. The type and amount of filler affect water absorption and surface wettability [17]. Modifying the surface layers can improve the durability of WPC composites based on lignocellulosic particles. The process of ion implantation as a surface modification method is a popular method used to improve the quality of many materials, mainly metals and their alloys. However, few research results indicate that ion implantation can also be a helpful method in modifying composites based on lignocellulosic particles in a polyethylene matrix. 

Knowing that in the process of ion implantation on the surface of various materials, it is possible to improve the properties of materials in a controlled and exact way by controlling the voltage and frequency, studies were undertaken to assess the effect of three fluencies of argon, 1 × 10^15^, 1 × 10^16^, and 1 × 10^17^ cm^−2^, at an accelerating voltage of 60 kV on the changes in wettability, surface energy, and colour of the WPC surface. The change in wettability or the increase in surface energy of hydrophobic materials such as PE can lead to many attractive solutions, e.g., absorbance on the surface of implanted materials of immiscible or non-reactive substances with polyethylene.

As a result, the ion implantation process could serve multiple beneficial functions. Firstly, it could induce structural changes, leading to the emergence of new and unconventional, yet intriguing, properties in a given material. These results could then be used to design production processes aimed at creating WPC composites with specific innovative properties, thereby demonstrating the practical value of the research in planning production processes.

## 2. Materials and Methods

### 2.1. WPC Preparation

WPC composites were prepared using high-density polyethylene (HDPE) (Hostalen GD 7255, Basell Orlen Polyolefins Sp. z o. o., Plock, Poland) and two types of lignocellulosic fillers: sawdust and conifer bark. Lignocellulosic material with a moisture content of 5% was ground and sorted into particles according to the guidelines described in previous publications [17,18]. The composite production process was identical to previous studies [18,19]. Table 1 describes the variants of the prepared composites. Two controls were used in this study: HDPE and recycled HDPE (Hostalen GD 7255, Basell Orlen Polyolefins Sp. z o. o., Plock, Poland).

### 2.2. Modelling and Ion Implantation

The ion implantation processes were preceded by the modelling (simulations) of the depth profiles of the implanted elements and their main parameters using SRIM-2013.00, the Stopping and Range of Ions in Matter (SRIM) freeware type code by James F. Ziegler (Annapolis, MD, USA) [20]. These parameters, such as maximum SRIM unit, *SRIMmax*, and associated with it peak volume dopant concentration, *Nmax*, projected range, *Rp*, range straggling, Δ*R_p_*, skewness, kurtosis, and sputtering yield, *Y* [21], are crucial in understanding the behaviour of the implanted ions and their interaction with the substrate.

The model also allows us to determine the value of the sputtering yield. However, this phenomenon in substrate sputtering by the implanted ions does not account for this phenomenon, similar to the substrate damage and the chemical reactions between the implanted ions and the substrate components.

The simulation was performed for 100,000 implanted ions perpendicular to the implanted substrate (the ion incidence angle was defined as 0°). In all cases, the simulations were performed for absolute zero.

The measured (densities) and estimated (quantities of the elements) values of the parameters for five types of implanted substrates were used. There were two kinds of pure polyethylene and three kinds of wood–plastic composites (WPCs), as follows:

- 100% polyethylene (HDPE);
- 100% recycled polyethylene (PE recycled);
- 40% sawdust + 60% HDPE;
- 50% sawdust + 50% HDPE;
- 60% sawdust + 40% HDPE.

The information about the end groups of polyethylene or the type of initiator used would be necessary for the precise calculations of the atomic percentage of each element. This percentage is 0.1–0.3% for a degree of polyethylene polymerisation of 1500. For simplicity, i.e., without considering the initiator and end groups, the polyethylene composition can be calculated using the mer formula (–CH_2_–CH_2_–). Its composition is about 33.33% carbon and 66.67% hydrogen in atomic percentage or 1 × 12.01 g C to 2 × 1.01 g H, which gives about 85.6% C and 14.4% H by weight.

The chemical composition of wood varies from species to species, but the central mass of wood consists of organic substances, which include four elements: carbon, hydrogen, oxygen, and nitrogen. On average, dry wood contains about 49.6% carbon, 6.3% hydrogen, and 44.2% oxygen, with nitrogen by weight. The nitrogen content in wood is, on average, 0.12%. In small quantities, the contents of other elements, like calcium, potassium, sodium, magnesium, iron, manganese, and additionally, sulfur, chlorine, silicon, and phosphorus, are about 1% by weight [22,23].

For simplicity, four main elements, carbon, hydrogen, oxygen, and nitrogen, were used in the model, with their total composition standardised to 100% by weight. In the next step, weight percentages were converted to atomic percentages used by the modelling code.

Finally, the chemical compositions of WPC were determined using simple Formulas (1)–(3), as follows:

(1) $${\%}_{X}=0.4{\%}_{X\mathrm{sawdust}}+0.6{\%}_{XPE}\;\text{for WPC of}\;40\%\;\text{sawdust}\;+\;60\%\;\text{PE}$$

(2) $${\%}_{X}=0.5{\%}_{X\mathrm{sawdust}}+0.5{\%}_{XPE}\;\text{for WPC of}\;50\%\;\text{sawdust}\;+\;50\%\;\text{PE}$$

(3) $${\%}_{X}=0.6{\%}_{X\mathrm{sawdust}}+0.4{\%}_{XPE}\;\text{for WPC of}\;60\%\;\text{sawdust}\;+\;40\%\;\text{PE}$$

where ${\%}_{X}$ is the total content of element *X* in WPC, ${\%}_{X\mathrm{sawdust}}$ is the content of element *X* in sawdust, and ${\%}_{XPE}$ is the content of element *X* in polyethylene.

Another simplification, based on the measurement results by Kraszkiewicz [24] presented in [24], concerned the difference in the content of the main elements in wood (sawdust) and tree bark. The difference in average carbon content in the bark compared to sawdust was only −0.8%. A similar situation was observed for hydrogen (−1.4%). The higher value was calculated for oxygen (−15.33%). Only the nitrogen content in the bark was more than 45-fold for the bark, but this difference was negligible due to the small total of this element. In other words, the proposed model does not distinguish composites into composites with sawdust and those with bark but generally calls them composites with sawdust.

The above-discussed final values of the element’s content used in the model, mainly rounded to two or three decimal places, are listed in Table 2. Additionally, the fundamental values of the substrate’s density have been shown.

Argon was used as the implanted element. Its atomic radius, which was determined from the minimal basis set SCF functions, is 0.71 Å [25].

The value of the acceleration voltage was 60 kV in all cases. This value, which was used in our previous experiments, is a typical value used in industry implanters. The projected range of the implanted ions is relatively wide in this case. This is important for heavier argon ions. Due to the single ionisation of the used ions, the values of the ion kinetic energy are numerically identical to the values of the accelerating voltage, i.e., 60 kV and 60 keV.

The implanted ions’ proposed fluencies were 1 × 10^15^, 1 × 10^16^, and 1 × 10^17^ cm^−2^ for all substrate cases. These used fluencies of the implanted ions (with the difference of 3 orders of magnitude) practically include the range of the fluencies used in industrial conditions.

**Figure 1:**
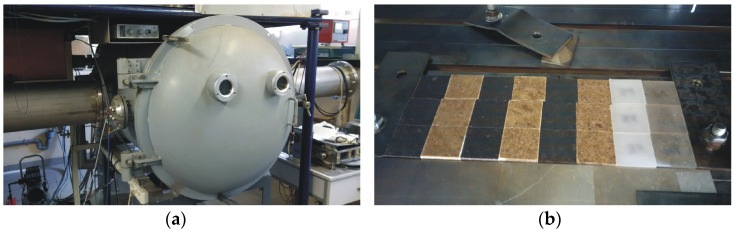
General view of the implanter of gaseous ion (**a**) and the implanted samples on XY table (**b**).

The ion implantation processes of argon were provided using a semi-industrial, non-mass-separated implanter of gaseous ions with a continuous ion beam (Figure 1a) used in the National Centre for Nuclear Research Świerk in Otwock, Poland, and described in detail elsewhere [16].

The implanted fluencies of argon were 1 × 10^15^, 1 × 10^16^, and 1 × 10^17^ cm^−2^. The acceleration voltage was 60 kV. The ion beam current was about 300 µA. The diameter of the ion beam was about 4 cm. Eight sets of the samples (HDPE, recycled HDPE, 40% sawdust + 60% HDPE, 40% bark + 60% HDPE, 50% sawdust + 50% HDPE, 50% bark + 50% HDPE, 60% sawdust + 40% HDPE, and 60% bark + 40% HDPE) with the dimensions of about 20 × 20 × 3 mm^3^ for sawdust reinforcement and about 20 × 20 × 2 mm^3^ for bark reinforcement were implanted on XY table (Figure 1b) for the increase in the implantation area and the decrease in the temperature of the sample surface. Additionally, the ion implantation with the highest fluence was provided in three stages to reduce the sample temperature. The time of the ion implantation of sample sets was about 77, 453 and 3 × 768 s (three stages of ion implantation processes) for the fluencies of 1 × 10^15^, 1 × 10^16^, and 1 × 10^17^ cm^−2^, respectively. The estimated temperature value of the implanted samples did not exceed 150 °C.

Argon of 99.999% purity, which was supplied by Kamino company (Warsaw, Poland), was used as a source of the implanted gaseous ions.

### 2.3. Determination of Colour Change

The colour parameters of the obtained samples were determined using the CIE Lab colour space model. Measurements were conducted with a Datacolor ColorReaderPRO spectrophotometer (Datacolor Technology, Suzhou, China), and the data were exported via the ColorReader App (Datacolor, Inc., Lawrenceville, NJ, USA). Each sample was measured in 10 repetitions.

The CIE Lab colour space model defines colour based on three parameters: lightness (L*), chromatic coordinate on the red-green axis (a*), and chromatic coordinate on the yellow–blue axis (b*). Lightness (L*), chromatic coordinates (a* and b*), and the total colour difference (ΔE) were measured for each sample according to the ISO 7724-3:2003 standard [26].

The total colour difference (ΔE) between samples was calculated using the following equation:

ΔE = [(ΔL)^2^ + (Δa)^2^ + (Δb)^2^]^1/2^, (4)

where ΔL = L_n_ − L_0_ is the difference in lightness between two measurements, Δa = a_n_ − a_0_ is the difference in the red-green coordinate, and Δb = b_n_ − b_0_ is the difference in the yellow–blue coordinate. Index 0 refers to a non-modified sample. Index n refers to the modified sample, where n = {1,2,3}, 1 is the sample implantation with a 1 × 10^15^ cm^−2^-ion dose, 2 is the sample implantation with a 1 × 10^16^ cm^−2^-ion dose, and 3 is the sample implantation with a 1 × 10^17^ cm^−2^-ion dose. 

The absolute colour difference (ΔE) was calculated to assess the colour change between measurements before and after specific treatments. The interpretation of ΔE values followed the following standard criteria:

0 < ΔE ≤ 1—unnoticeable difference;
1 < ΔE ≤ 2—difference noticed by an experienced observer;
2 < ΔE ≤ 3.5—difference noticed by an inexperienced observer;
3.5 < ΔE ≤ 5—noticeable difference;
5 < ΔE—significant colour change.

### 2.4. Determination of the Wetting Angle and Surface Free Energy

The contact angle for the prepared samples of each variant was determined using a Haas Phoenix 300 goniometer (Surface Electro Optics, Suwon City, Republic of Korea). The measurements were performed using water as a polar liquid and diiodomethane as a non-polar liquid. A precision syringe deposited approximately 5 µL of liquid onto the sample’s surface. The goniometer was equipped with lenses and a digital camera. Image XP analysis software (Surface Electro Optics, version 5.8, Suwon City, Republic of Korea) was used to capture images of the droplets on the sample surfaces and calculate the contact angles by analysing the contour of the drops. The contact angle measurements were taken at 5, 20, 40, and 60 s after placing a droplet on the sample surface.

Each measurement was repeated five times, and the mean contact angle values were used for further analysis. The surface free energy of the samples was then calculated using the Owens–Wendt method, which utilises the contact angles obtained from both water and diiodomethane. Calculations were performed automatically using the Image XP analysis software.

### 2.5. Statistical Analysis

A statistical analysis of the results was carried out in Statistica (data analysis software system) version 13 (TIBCO Software Inc., Palo Alto, CA, USA). An analysis of variance (MANOVA) was used to test (significant level = 0.05) for significant differences between factors. A comparison of the means was performed using Tukey’s test, with a 0.05 significance level.

## 3. Results and Discussion

### 3.1. Modelling and Ion Implantation Results

**Figure 2:**
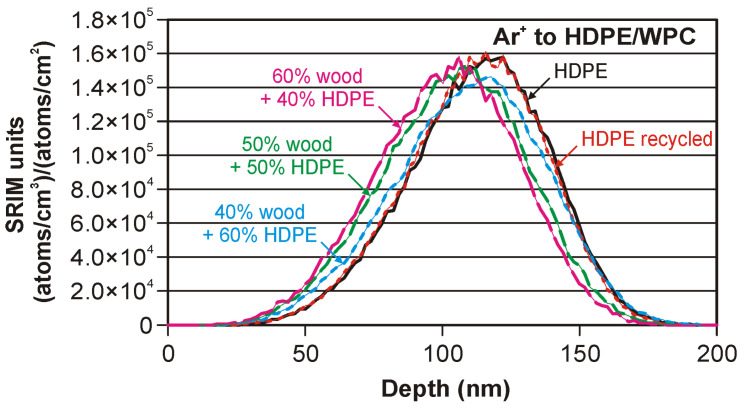
Modelled depth profiles for two kinds of polyethylene and three kinds of wood–plastic composites.

Figure 2 presents the results of the computer model of the depth profiles for argon ions implanted in five kinds of substrate. 

Table 3 shows the values of the maximum SRIM unit, *SRIM_max_*, and the peak volume dopant concentration associated with it, *N_max_*, for the fluences of 1 × 10^15^, 1 × 10^16^, and 1 × 10^17^ cm^−2^, projected range, *R_p_*, range straggling, Δ*R_p_*, skewness, kurtosis, total sputtering yield, *Y_total_*, and sputtering yield for four main elements of the substrates. The percentage values of relative difference from PE are presented in round brackets.

The presented “SRIM units” in (atoms/cm^3^)/(atoms/cm^2^) are special units of plot ordinate used in SRIM code results. With these units multiplied by the ion fluence (in atoms/cm^2^), the ordinate values convert directly into a density distribution with the unit of atoms/cm^3^.

The results in the model suggest that the modified region is thinner than 200 nm in each considered case.

The depth profiles of argon are very similar for both polyethylene substrates (HDPE and recycled HDPE). The density change from 0.867 to 0.874 g/cm^2^ does not visibly change the depth profile’s position and shape. In this case, the difference for all main peak parameters and the sputtering yield is less than 2.5%.

Significant differences are observed for WPC substrates. The values of the projected range are smaller for the composites with a higher sawdust content. The calculated change is 2.68, 7.78, and 10.2% for 40, 50, and 60% wood, respectively. The values of the range straggling change from 1.58 to 7.11%. The kurtosis change is at the level of 1.6–2.6%. The highest change is observed for the skewness. This parameter changes from about 26% to about 42%. The total sputtering yield changes in the range of 9.14–13.65%. The change values for carbon are several times larger than for hydrogen.

The ion implantation damages the surface of the implanted substrates for the proposed implantation parameters, especially for the highest fluence. Visually, the damage is higher for the bark reinforcement. It seems that the amount of reinforcement is less significant.

### 3.2. Colour Change Results

**Figure 3:**
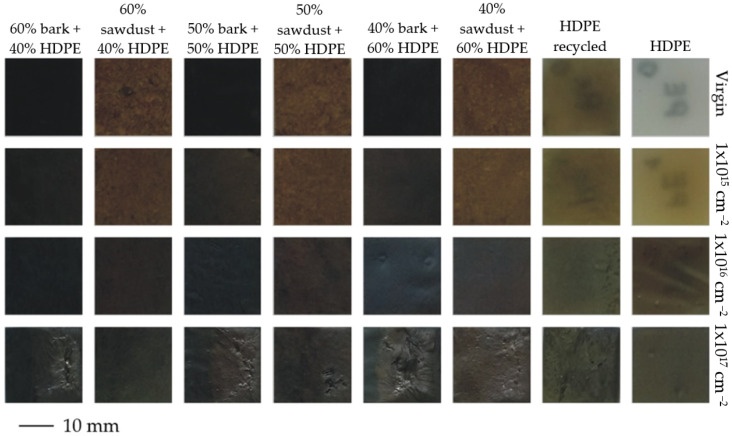
Naked-eye observation results of the surface of the virgin and the implanted samples.

Figure 3 presents the naked-eye observation results of the virgin surface and the implanted samples. Unfortunately, the markings of the four HDPE samples (virgin HDPE, 1 × 10^15^ HDPE, recycled virgin HDPE, and 1 × 10^15^ recycled HDPE) are visible from the tested side. The non-implanted HDPE and recycled HDPE control samples and those implanted with a beam fluence of 1 × 10^15^ cm^−2^ remain semi-transparent. 

Implantation affects the colour change due to covering the sample with a layer of implanted ions (Table 4). This change is evident in each case compared to the variant without implantation (5 < ΔE—the observer has the impression of two different colours). The introduction of implantation also affects the colour balance between the individual variants. The differences in the colour of the samples before implantation, especially in the range of lightness (L*), are more pronounced (Table 4). It can also be seen that ion implantation generates colour changes on the surface of samples containing cortex as a filler towards green and blue shades, which is not usually observed in the case of WPCs containing sawdust as a filler. Argon ion implantation also leads to a change in the chromatic coordinates of pure polyethylene. The HDPE surface exposed to argon ions was found to be characterised by a decrease in lightness and a colour change toward shades of yellow and red (Table 4).

The observed differences among the chromatic coordinate values depend mainly on the raw material composition of the WPC. According to Persze and Tolvaj [27], chromophore groups in wood occur mainly in lignin and are responsible for the change in the colour of lignocellulosic particles. Additionally, implantation of the material surface with argon ions causes lattice disorder, which may manifest itself in optical changes [28]. In the patent US 7,604,846 B2 [29], the authors demonstrated that ion implantation is a method for imparting permanent colours to diamonds, and the conditions of ion implantation and additional heat treatment impact the permanent effect of colour development. Chen et al. [30] indicate that colour changes in materials subjected to implantation processes may be related to the change in the valence of elements and the existence of oxygen vacancies. In turn, Rao et al. [31] postulate that irradiation of materials with an ion beam is a valuable method for fine-tuning colours, such as toning or hue development, in which the choice of ions, energy, and fluence are important. 

### 3.3. Wetting Angle and Surface Free Energy Results

**Figure 4:**
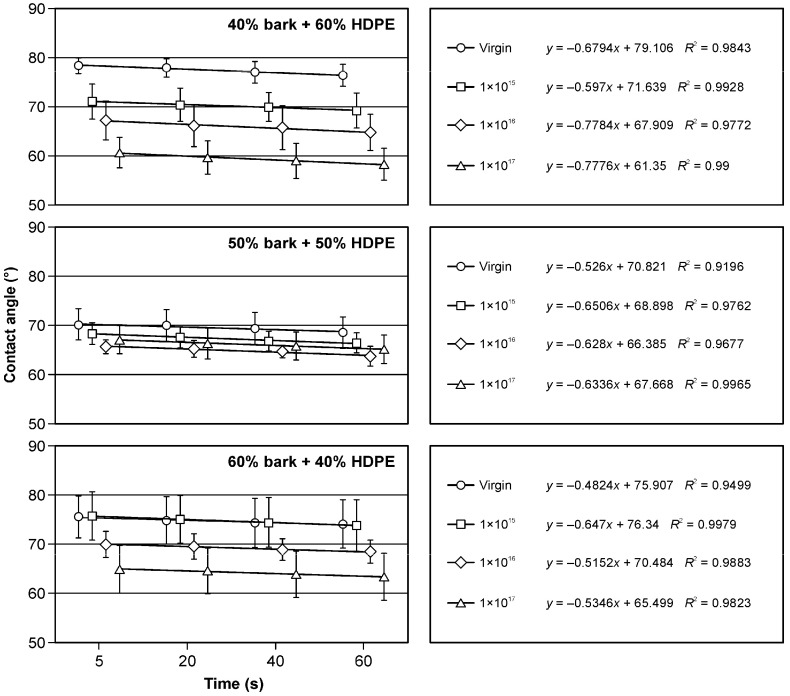
Changes in the wetting angle values of HDPE- and bark-based WPC composite surfaces depending on the argon ion implantation dose.

**Figure 5:**
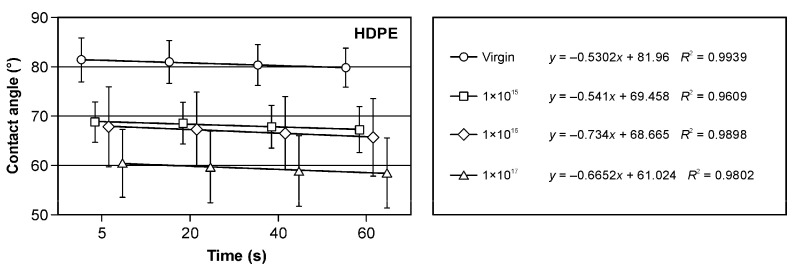
Changes in the wetting angle of the polyethylene surface depending on the argon ion implantation dose.

**Figure 6:**
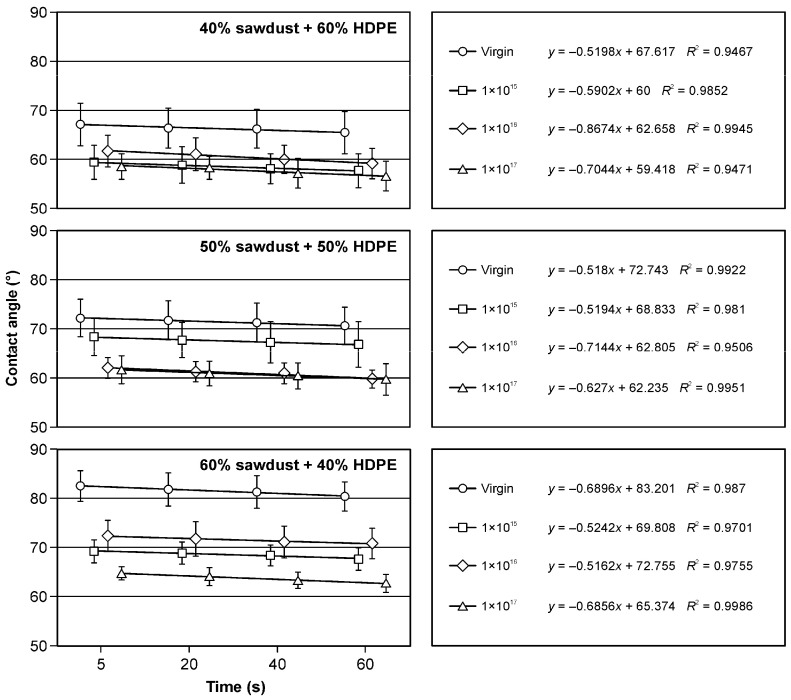
Changes in the wetting angle values of HDPE- and sawdust-based WPC composite surfaces depending on the argon ion implantation dose.

The implantation of pure polyethylene and samples containing fillers in the form of sawdust and bark with argon ions has practical implications. The decrease in the wetting angle (Figure 4, Figure 5 and Figure 6) is a crucial observation. Notably, the presence of fillers in the form of bark in the amounts of 50% and 60% influenced the increase in surface wettability (Figure 4) in comparison to samples of pure HDPE (Figure 5) and those containing 40% of filler in the form of bark (Figure 4). The wettability of non-implanted samples containing 40% of bark was similar to that of pure, non-implanted polyethylene (Figure 5). In most of the analysed research variants, the most significant influence on the changes in the wetting angle was the ion fluence of 1 × 10^17^ cm^−2^. Only in the case of the variant containing 50% filler in the form of bark was a higher surface wettability observed at the argon ion fluence of 1 × 10^16^ cm^−2^ (Figure 4). Observing changes in surface wettability over time from 5 to 60 s, a slight decrease in the wetting angle was observed in all the variants tested. In none of the analysed cases did this change exceed 4%. These findings directly affect the design and engineering of materials with specific surface properties.

Also, the filler in the form of sawdust in the amount of 40 and 50% caused a decrease in the surface wetting angle (Figure 6) compared to pure polyethylene (Figure 5), which was visible in the non-implanted samples. In the case of the filler, the share of which was 60% (Figure 6), a similar value of the wetting angle of the non-implanted surface was observed compared to the control. Also, in these research variants, a decrease in the wetting angle of the surfaces subjected to implantation with argon ions was observed. The changes in the surface wettability of the samples containing fillers in the form of sawdust occurring from 5 to 60 s were comparable to those observed for the samples containing bark as a filler.

The tests presented in Table 5 indicate an increase in the surface free energy due to implanting the composite surface with argon ions. The highest values were recorded for the WPC variant, on the surface of which the argon ion fluence was 1 × 10^17^ cm^−2^ (regardless of the content and type of filler). In contrast, the lowest values were characteristic of the composite surface, which was not modified with argon ions. The differences in the surface free energy values within the individual composite variants were the smallest in the case of WPC variants on the surface, of which the argon ion fluence was 1 × 10^17^ cm^−2^ and the largest for the composites not modified with argon ions. The filler content in the form of sawdust had the slightest effect on the changes in the surface free energy value for the WPC variants on the surface, of which the argon ion fluence was 1 × 10^15^ cm^−2^. The opposite relationship was observed for the composites containing sawdust filler, on whose surface the argon ion fluence was 1 × 10^16^ cm^−2^.

Regarding the analysis of the surface free energy value, it should be noted that the physicochemical parameters of the surface directly affect the way it interacts with factors such as water or organic solvents, which consequently translates into resistance or a lack thereof to factors that degrade a given material. One of the essential tools for describing the physicochemical properties of the surface of a solid is the wetting angle. Based on its value (depending on the type of reference liquid used), the surface free energy is also determined, among other things. It is a parameter that directly determines the interaction of the substrate with agents such as varnish or glue. Without proper physicochemical characterisation of the material surface, it is difficult to determine its functional properties. Both the wetting angle and the surface free energy determine the functional and processing properties of the material, such as the susceptibility to glueing, refinement, or impregnation with agents, e.g., fire retardants or those protecting against biological corrosion. It is generally known that the higher the value of the surface free energy, the more wettable the material—the more hygroscopic. For example, according to de Meijer et al. [32] and Gérardin et al. [33], the values of the surface free energy for raw wood, depending on the species, are as follows: pine: 83.8 mJ/m^2^ (early wood)—71.1 mJ/m^2^ (late wood); beech: 58.5 mJ/m^2^; and oak: 45.11 mJ/m^2^. For comparison, the value of the parameter determined for Teflon (difficult to wet substrate) is only 18 mJ/m^2^. It should be noted that regarding the results of the surface free energy tests of the produced materials obtained in this work, the obtained values confirm the previous literature reports [11] and confirm that the implantation of Ar ions on the surface of materials containing plastics translates into changes in the wettability of the surface of these materials.

### 3.4. Statistical Analysis of Results

Table 6 presents the analysis of the percentage influence of individual variable factors (filler type, filler amount, implanted fluencies of argon, time after placing a droplet) and interactions between these factors on the values of surface wetting angles. It should be noted that all the analysed factors showed a statistically significant influence (*p* < 0.05), while interactions between these factors in most cases did not have a statistically significant influence (*p* > 0.05) on the value of the wetting angle. In general, it can be stated that among the analysed variable factors, the implanted fluencies of argon (X = 45.07%) had the most significant percentage influence on the value of the wetting angle. This was the dominant factor. Among the material factors, the filler amount (X = 11.01%) showed the most significant percentage influence on the value of the wetting angle. In turn, the percentage influence of the filler type was only 2.07%. It is worth pointing out here that the percentage impact of factors not taken into account during the research was much higher and amounted to error = 24.76%.

## 4. Conclusions

In this study, we implanted the surface of WPC composites with a beam of Ar ions with fluences of 1 × 10^15^, 1 × 10^16^, and 1 × 10^17^ cm^−2^. This study aimed to check how the surface properties of the composites change, such as the wetting angle, surface free energy, and colour.

- During this study, it was found that the most significant influence on the analysed features is exerted by the dose of ions and the share of lignocellulosic raw material in the composition of the composites;
- Studies of the ion penetration depth profile indicate that the layer on which the changes occur reaches 200 nm. Additionally, the surface is damaged, which was visible, especially at the highest doses used;
- It seems that surface damage can be minimised through modification with a lower-current ion beam;
- The implantation of Ar ions on the WPC surface led to a slight decrease in the wetting angle and an increase in the free surface energy compared to non-implanted samples.

Differences in wetting angle and surface energy can have a significant impact on the properties of composites. They can play a crucial role in changing the adhesive properties of surfaces. With appropriately selected modification parameters, the adhesive properties of WPC can be significantly improved. This potential for improvement is an inspiring aspect of our research, especially in designing WPC composites with improved functional features.

## Figures and Tables

**Table 1:** Characteristics of the composition of raw materials used for the production of WPC.

Samples	HDPE (%)	Sawdust (%)	Bark (%)
40% sawdust + 60% HDPE	60	40	-
40% bark + 60% HDPE	60	-	40
50% sawdust + 50% HDPE	50	50	-
50% bark + 50% HDPE	50	-	50
60% sawdust + 40% HDPE	40	60	-
60% bark + 40% HDPE	40	-	60
Recycled HDPE	100	-	-
HDPE	100	-	-

**Table 2:** Values of the atomic percentage and densities of the substrates used in the model.

Substrate	at.% C	at.% H	at.% O	at.% N	Density (g/cm^3^)
HDPE	33.33	66.67	-	-	0.867
Recycled HDPE	33.33	66.67	-	-	0.874
40% sawdust + 60% HDPE	32.56	59.02	8.38	0.03	0.977 *
50% sawdust + 50% HDPE	32.37	57.11	10.48	0.03	1.052 *
60% sawdust + 40% HDPE	32.18	55.2	12.58	0.04	1.100 *

* Values for the WPC with sawdust.

**Table 3:** Values of the peak parameters of the model of the implanted ions in five kinds of substrate.

Parameter	HDPE	HDPE Recycled	40% Sawdust + 60% HDPE	50% Sawdust + 50% HDPE	60% Sawdust + 40% HDPE
*SRIM_max_* (atoms/cm^3^)/(atoms/cm^2^)	1.58 × 10^5^	1.61 × 10^5^ (2%)	1.47 × 10^5^ (−7.1%)	1.52 × 10^5^ (−3.49%)	1.58 × 10^5^ (0.25%)
*N_max_* (cm^−3^)	1.58 × 10^20^ 1.58 × 10^21^ 1.58 × 10^22^	1.61 × 10^20^ (2%) 1.61 × 10^21^ (2%) 1.61 × 10^22^ (2%)	1.47 × 10^20^ (−7.1%) 1.47 × 10^21^ (−7.1%) 1.47 × 10^22^ (−7.1%)	1.52 × 10^20^ (−3.49%) 1.52 × 10^21^ (−3.49%) 1.53 × 10^22^ (−3.49%)	1.58 × 10^20^ (0.25%) 1.58 × 10^21^ (0.25%) 1.58 × 10^22^ (0.25%)
*R_p_* (nm)	111.8	110.9 (−0.81%)	108.8 (−2.68%)	103.1 (−7.78%)	100.4 (−10.2%)
Δ*R_p_* (nm)	50.6	50.2 (−0.79%)	54.2 (7.11%)	52.2 (3.16%)	51.4 (1.58%)
Skewness	−0.2337	−0.2391 (2.31%)	−0.1718 (−26.49%)	−0.1445 (−38.17%)	−0.1353 (−42.11%)
Kurtosis	2.8273	2.8392 (0.42%)	2.7826 (−1.58%)	2.7531 (−2.62%)	2.7531 (−2.62%)
*Y_C_* (atoms/ion)	0.1863	0.1855 (−0.43%)	0.2167 (16.32%)	0.2235 (19.97%)	0.2313 (24.15%)
*Y_H_* (atoms/ion)	1.16	1.15 (−0.86%)	1.11 (−4.31%)	1.09 (−6.03)	1.07 (−7.76%)
*Y_O_* (atoms/ion)	-	-	0.1421	0.183	0.228
*Y_N_* (atoms/ion)	-	-	0.00054	0.00049	0.00071
*Y_total_* (atoms/ion)	1.3463	1.3355 (−0.8%)	1.46934 (9.14%)	1.49699 (11.19%)	1.53001 (13.65%)

**Table 4:** Comparison of spectrophotometric measurements on the WPC surface before and after argon ion implantation.

Filler Type	Filler Amount (%)	Fluence (cm^−2^)	L*	a*	b*	ΔE
bark	0	0	52.046 (±3.355)	1.878 (±1.695)	−4.021 (±2.084)	-
	40		26.096 (±0.291)	1.680 (±0.483)	1.713 (±0.367)	
	50		24.180 (±0.410)	1.664 (±0.372)	1.980 (±0.496)	
	60		25.024 (±0.519)	0.705 (±0.314)	0.904 (±0.400)	
sawdust	0	0	52.046 (±3.355)	1.878 (±1.695)	−4.021 (±2.084)	-
	40		46.631 (±0.981)	7.375 (±0.841)	20.087 (±0.750)	
	50		47.980 (±1.308)	8.929 (±1.336)	18.996 (±1.582)	
	60		43.946 (±0.641)	8.644 (±2.383)	19.233 (±1.152)	
bark	0	1 × 10^15^	48.174 (±1.880)	0.255 (±1.140)	9.003 (±0.742)	13.684
	40		34.216 (±0.168)	0.184 (±0.306)	2.979 (±0.475)	8.353
	50		36.940 (±0.546)	1.939 (±0.442)	5.326 (±0.560)	13.194
	60		36.285 (±0.401)	1.944 (±0.297)	4.672 (±0.720)	11.939
sawdust	0	1 × 10^15^	48.174 (±1.880)	0.255 (±1.140)	9.003 (±0.742)	13.684
	40		47.808 (±0.562)	6.013 (±1.121)	18.692 (±0.896)	2.227
	50		47.594 (±0.401)	7.056 (±0.501)	17.498 (±0.412)	2.429
	60		45.138 (±0.422)	7.152 (±0.958)	16.585 (±0.612)	3.269
bark	0	1 × 10^16^	43.573 (±0.742)	1.510 (±1.309)	1.692 (±0.807)	10.226
	40		39.417 (±0.216)	−1.346 (±0.941)	−0.71 (±0.579)	13.874
	50		37.687 (±0.460)	−0.839 (±0.551)	−1.009 (±0.505)	14.085
	60		38.793 (±0.323)	−0,405 (±1.096)	−1.622 (±0.636)	14.043
sawdust	0	1 × 10^16^	43.573 (±0.742)	1.510 (±1.309)	1.692 (±0.807)	10.226
	40		42.535 (±0.183)	2.309 (±0.321)	3.529 (±0.707)	17.794
	50		43.181 (±0.232)	4.263 (±1.193)	4.238 (±0.920)	16.205
	60		41.786 (±0.181)	3.456 (±0.438)	3.631 (±0.491)	16.583
bark	0	1 × 10^17^	45.318 (±0.193)	2.327 (±0.314)	3.681 (±0.226)	10.237
	40		42.815 (±2.733)	−0.317 (±3.904)	0.791 (±2.398)	16.863
	50		40.361 (±4.162)	3.393 (±1.887)	2.001 (±1.687)	16.273
	60		44.733 (±2.138)	2.751 (±2.493)	0.461 (±1.360)	19.820
sawdust	0	1 × 10^17^	45.318 (±0.193)	2.327 (±0.314)	3.681 (±0.226)	10.237
	40		44.933 (±1.175)	3.243 (±0.829)	2.992 (±0.427)	17.669
	50		43.396 (±0.948)	−0.437 (±1.554)	5.759 (±1.972)	16.851
	60		42.299 (±1.803)	2.272 (±1.089)	3.459 (±0.948)	17.092

**Table 5:** Values of surface free energy.

Filler Type	Filler Amount (%)	Fluence (cm^−2^)			
		0	1 × 10^15^	1 × 10^16^	1 × 10^17^
		Surface Free Energy (mJ/m^2^)			
bark	0	44.5	51.4	51.1	54.4
	40	44.6	49.9	51.8	55.3
	50	46.9	52.0	53.1	52.4
	60	48.1	48.0	50.3	54.0
sawdust	0	44.5	51.4	51.2	54.4
	40	48.9	56.7	55.7	56.1
	50	46.6	51.8	55.4	54.5
	60	43.8	51.5	48.8	52.8

**Table 6:** Analysis of variance of surface contact angle measurement results.

Factors	*p*	X (%)
Filler type (A)	1.39 × 10^−10^	2.07
Filler amount (B)	<1.00 × 10^−17^	11.01
Implanted fluencies of argon (C)	<1.00 × 10^−17^	45.07
Time after placing a droplet (D)	5.89 × 10^−4^	0.85
A × B	<1.00 × 10^−17^	4.51
A × C	6.08 × 10^−4^	0.85
B × C	<1.00 × 10^−17^	6.40
A × D	1.00	<0.01
B × D	1.00	0.01
C × D	1.00	0.01
A × B × C	1.61 × 10^−14^	4.44
A × B × D	1.00	<0.01
A × C × D	1.00	<0.01
B × C × D	1.00	0.01
A × B × C × D	1.00	0.01
Error		24.76

*p*—significant with α = 0.05; X—percentage of contribution.

## Data Availability

The original contributions presented in the study are included in the article, further inquiries can be directed to the corresponding authors.

## References

[1] Li L., Zhang Z., Zhang D., Qi F., Dai Y., Wei W., Ouyang X. (2024). Effects of metal ion implantation (Fe, Ti, Zn and Zr) on mechanical properties, corrosion resistance and biocompatibility of WE43 Mg alloy. J. Magnes. Alloy..

[2] Bai W., Zhou Y., Xiao M., Xu L., Xiao H., Tong Y., He C., Pang J., Xie Q., Yang C. (2024). Effects of Cu ion implantation on the microstructure, dielectric and impedance properties of SrTiO_3_ ceramics prepared by reduction-reoxidation method. Ceram. Int..

[3] Williams J.S. (1998). Ion implantation of semiconductors. Mater. Sci. Eng. A.

[4] Resende R.C., Ribeiro R.P., Waldman W.R., Cruz N.C., Araujo J.R., Rangel E.C. (2020). Improvement of thermoplastic elastomer degradation resistance by low-energy plasma immersion ion bombardment. Mater. Chem. Phys..

[5] Karthikeyan K.R., Thanigai Arul K., Ramana Ramya J., Nabhiraj P.Y., Menon R., Krishna J.B.M., Narayana Kalkura S. (2020). Novel microporous surface and blue emission of argon ion implanted polyvinylacohol/bionanohydroxyapatite coatings. Radiat. Phys. Chem..

[6] Prakrajang K., Wanichapichart P., Anuntalabhochai S., Pitakrattananukool S., Yu L.D. (2009). Ion beam modification of chitosan and cellulose membranes for simulation of ion bombardment of plant cell envelope. Nucl. Instrum. Methods Phys. Res. B Beam Interact. Mater. At..

[7] Barlak M., Wilkowski J., Werner Z. (2016). Ion implantation changes of tribological and corrosion resistance properties of materials used in wood industry. Ann. Wars. Univ. Life Sci.-SGGW For. Wood Technol..

[8] Barlak M., Wilkowski J., Boruszewski P., Werner Z., Pałubicki B. (2017). Changes of functional properties of materials used in wood industry after ion implantation processes. Ann. Wars. Univ. Life Sci.-SGGW For. Wood Technol..

[9] Wilkowski J., Barlak M., Bottger R., Werner Z., Konarski P., Pisarek M., Wachowicz J., Von Borany J., Auriga A. (2021). Effect of nitrogen ion implantation on the life time of WC-Co tools used in particleboard milling. Wood Mater. Sci. Eng..

[10] Kondyurin A., Gan B.K., Bilek M.M.M., McKenzie D.R., Mizuno K., Wuhrer R. (2008). Argon plasma immersion ion implantation of polystyrene films. Nucl. Instrum. Methods Phys. Res. B Beam Interact. Mater. At..

[11] Sun C., Sprouster D.J., Zhang Y., Chen D., Wang Y., Ecker L.E., Gan J. (2019). Formation window of gas bubble superlattice in molybdenum under ion implantation. Phys. Rev. Mater..

[12] Narula C., Chauhan R.P., Garg A., Rana P., Panchal S., Gupta R. (2024). Modification of the Properties of CdSe Nanowires by Argon Ion Implantation. J. Electron. Mater..

[13] Lu N., Chen Z., Zhang W., Yang G., Liu Q., Böttger R., Zhou S., Liu Y. (2021). Effect of silver ion implantation on antibacterial ability of polyethylene food packing films. Food Packag. Shelf Life.

[14] Shanthini G.M., Sakthivel N., Menon R., Nabhiraj P.Y., Gómez-Tejedor J.A., Meseguer-Dueñas J.M., Gómez Ribelles J.L., Krishna J.B.M., Narayana Kalkura S. (2016). Surface stiffening and enhanced photoluminescence of ion implanted cellulose—Polyvinyl alcohol—Silica composite. Carbohydr. Polym..

[15] Chan K.F., Ho J.P.Y., Li W.Y., Lau B.M.F., Tse A.K.W., Fong W.F., Bilek M.M.M., McKenzie D.R., Chu P.K., Yu K.N. (2007). Investigation of cytocompatibility of surface-treated cellulose nitrate films by using plasma immersion ion implantation. Surf. Coat. Technol..

[16] Betlej I., Barlak M., Wilkowski J., Werner Z., Zagórski J., Lipska K., Boruszewski P. (2022). Wettability of the surface of bacterial cellulose film modified with the ion implantation. Ann. Wars. Univ. Life Sci.-SGGW. For. Wood Technol..

[17] Chmielnicki B., Konieczny J. (2014). Properties of WPC composites with polyethylene matrix filled with nutshell flour. Przetwórstwo Tworzyw.

[18] Borysiuk P., Auriga R., Wilkowski J., Auriga A., Trocinński A., Seng Hua L. (2022). A Study on the Susceptibility of PLA Biocomposites to Drilling. Forests.

[19] Borysiuk P., Boruszewski P., Auriga R., Danecki L., Auriga A., Rybak K., Nowacka M. (2021). Influence of a bark-filler on the properties of PLA biocomposites. J. Mater. Sci..

[20] SRIM. http://www.srim.org/.

[21] Barlak M., Wilkowski J., Szymanowski K., Czarniak P., Podziewski P., Werner Z., Zagórski J., Staszkiewicz B. (2019). Influence of the ion implantation of nitrogen and selected metals on the lifetime of WC-Co indexable knives during MDF machining. Ann. Wars. Univ. Life Sci.-SGGW. For. Wood Technol..

[22] Otwarta Encyklopedia Leśna. https://www.encyklopedia.lasypolskie.pl/doku.php?id=b:budowa-i-sklad-chemiczny-drewna.products.

[23] Barette J.P., Hazard C., Mayer J. (1996). Mémotech—Bois et Matériaux Associés.

[24] Kraszkiewicz A. (2009). Analiza wybranych właściwości chemicznych drewna i kory robinii akacjowej (*Robinia pseudoacacia* L.). Inżynieria Rol..

[25] Clementi E., Raimondi D.L., Reinhardt W.P. (1967). Atomic screening constants from SCF functions. II. Atoms with 37 to 86 electrons. J. Chem. Phys..

[26] (2003). Paints And Varnishes—Colorimetry—Part 3: Calculation of Colour Differences.

[27] Persze L., Tolvaj L. (2012). Photodegradation of wood at elevated temperature: Colour change. J. Photochem. Photobiol. B Biol..

[28] Yang H., Matinrad H., Goldbaum M.H., Bu J.J., Fukuoka H., Afshari N.A. (2023). Refractive Changes After Implantation of Reversed Intraocular Lens in Cataract Surgery: A Mathematical Model. J. Refract. Surg..

[29] Park J., Lee J., Sohn C., Choi B. (2009). Manufacturing Method of Colored Damond by on Miplantation and Heat Treatment. U.S. Patent.

[30] Chen R., Lu W., Lu J., Pu R., Lin J., Yu J. (2022). The color mechanism of iron on quartz by ion implantation. Phys. B Condens. Matter.

[31] Rao K.S., Sahoo R.K., Dash T., Magudapathy P., Panigrahi B.K., Nayak B.B., Mishra B.K. (2016). N and Cr ion implantation of natural ruby surfaces and their characterization. Nucl. Instrum. Methods Phys. Res. B Beam Interact. Mater. At..

[32] de Meijer M., Haemers S., Cobben W., Militz H. (2000). Surface Energy Determinations of Wood: Comparison of Methods and Wood Species. Langmuir.

[33] Gérardin P., Petrič M., Petrissans M., Lambert J., Ehrhrardt J.J. (2007). Evolution of wood surface free energy after heat treatment. Polym. Degrad. Stab..

